# Editorial: Safety of Polyphenolic Compounds and Their Role in Cardiovascular Diseases

**DOI:** 10.3389/fcvm.2022.940967

**Published:** 2022-06-24

**Authors:** Lamiaa A. Ahmed, Rabab H. Sayed, Wafaa R. Mohamed, El-Shaimaa A. Arafa

**Affiliations:** ^1^Department of Pharmacology and Toxicology, Faculty of Pharmacy, Cairo University, Cairo, Egypt; ^2^Department of Pharmacology and Toxicology, Faculty of Pharmacy, Beni-Suef University, Beni-Suef, Egypt; ^3^Department of Clinical Sciences, College of Pharmacy and Health Sciences, Ajman University, Ajman, United Arab Emirates; ^4^Center of Medical and Bio-Allied Health Sciences Research, Ajman University, Ajman, United Arab Emirates

**Keywords:** antioxidants, ischemic heart diseases, oxidative stress, quercetin, resveratrol

Oxidative stress plays a critical role in the pathophysiology of various ischemic heart diseases (IHDs) including coronary artery disease and myocardial infarction, and their associated complications such as arrhythmia, heart failure, cardiogenic shock, and sudden cardiac arrest. Additionally, various conditions linked to IHDs are associated with excessive oxidative stress and/or depletion of endogenous antioxidant defense mechanisms including diabetes and hyperlipidemia. Therefore, it would be rational to use antioxidants as complementary therapies in these settings. Polyphenolic compounds have been used in ischemic cardiovascular diseases (CVDs) that involve alteration of the redox cellular equilibrium. These compounds include gallic acid, vanillic acid, ellagic acid, caffeic acid, quercetin, rutin, kaempferol, catechin, and epicatechin.

The current Research Topic served as a domain to publish research and review articles on the use of polyphenolic compounds in the management of IHDs and their complications with emphasis on their protective potentials and the underlying mechanisms where some of these studies were published in this Research Topic as clarified here.

Quercetin is a natural flavonoid found in many fruits and vegetables. Growing evidences suggest that quercetin could lower the risk of IHDs by improving endothelial dysfunction and its associated risk factors, such as hypertension and atherosclerosis. The review article published by Dagher et al. summarized some of the experimental and clinical trials revealing its cardiovascular effects. They discussed the prospect for the clinical use of quercetin as a nutraceutical against endothelial dysfunction in IHDs, suggesting a great need for well-designed clinical trials to confirm its beneficial effects. The data presented by this review article were further emphasized by the investigation performed by Dagher et al. which revealed that quercetin supplementation could limit the inflammatory response triggered by coronary artery bypass graft operation and thus could prevent the post-operative complications. This study suggested that quercetin could improve endothelial function by eliminating senescent vascular endothelial cells. These results provided valuable information regarding a novel approach to improve clinical outcomes through the use of a safe and a natural agent as an adjuvant therapy in the management of acute coronary syndrome.

Another review article by Chedea et al. discussed the antioxidant and pro-oxidant effects of resveratrol and polyphenolic grapevine byproducts assessed in *in vitro* or *in vivo* IHDs conditions. These studies revealed that the antioxidant/pro-oxidant balance after administration of these compounds is dose and time dependent. Thus, choosing the optimal dose and time for administration of these compounds may have a positive impact on maintaining the redox endothelial balance and controlling atherosclerosis development. Knowing that endothelial dysfunction is a condition that precedes the clinically manifested IHDs, these by-products, especially grape pomace, grape seed, and resveratrol could be used in patients with high cardiovascular risks.

Furthermore, Bustos et al.'s review provided an overview of *in vitro* and *in vivo* studies demonstrating the effect of different polyphenolic compounds in the prevention of post-kidney transplantation injuries and the associated cardiovascular complications. These outcomes could be in part through the counterbalance of oxidative stress and inflammation and thus the prevention of metabolic complications that increase cardiovascular risk. Bustos et al. suggested that more observational and interventional clinical studies are required to emphasize the direct effect of polyphenolic compounds in kidney transplant recipients and their impact on the prevention of the associated chronic complications such as CVDs.

Therefore, the current Research Topic revealed the safety and efficacy of these polyphenolic compounds in well-organized studies in various ischemic cardiovascular conditions. We hope that these data will guide more researchers by providing new directions for the early prevention and management of IHDs.

## Author Contributions

All authors listed have made a substantial, direct, and intellectual contribution to the work and approved it for publication.

## Conflict of Interest

The authors declare that the research was conducted in the absence of any commercial or financial relationships that could be construed as a potential conflict of interest.

## Publisher's Note

All claims expressed in this article are solely those of the authors and do not necessarily represent those of their affiliated organizations, or those of the publisher, the editors and the reviewers. Any product that may be evaluated in this article, or claim that may be made by its manufacturer, is not guaranteed or endorsed by the publisher.

